# Resilience and associated factors among Chinese patients diagnosed with oral cancer

**DOI:** 10.1186/s12885-019-5679-0

**Published:** 2019-05-14

**Authors:** Yuqin Gao, Lulu Yuan, Bochen Pan, Lie Wang

**Affiliations:** 1 Department of Nursing, School of Stomatology, China Medical University, Shenyang, People’s Republic of China; 2 Shengjing Hospital, China Medical University, Shenyang, People’s Republic of China; 3 School of Public Health, China Medical University, Shenyang, People’s Republic of China

**Keywords:** Oral cancer, Resilience, Hope, Optimism, Positive psychology

## Abstract

**Background:**

Resilience has been linked to psychological adaptation to many challenging life events. The present study aims to explore the level of resilience in oral cancer patients and the key factors associated with resilience, and to evaluate the relationship between resilience and anxiety.

**Methods:**

A multiple center cross-sectional study was carried out for Chinese patients with oral cancer between May 2016 and October 2017 in the Stomatology Hospital of China Medical University and Department of Stomatology, Shengjing Hospital of China Medical University. Two hundred and thirty oral cancer patients replied to the questionnaires on resilience, hope, perceived social support, optimism, perceived stress and anxiety which were measured with Resilience Scale-14 (RS-14), Herth Hope Index (HHI), Multidimensional Scale of Perceived Social Support (MSPSS), Revised Life Orientation Test (LOT-R), Perceived Stress Scale-10 (PSS-10) and Zung Self-Rating Anxiety Scale (SAS), respectively. Univariate one-way ANOVA/t-test, Pearson’s r and hierarchical linear regression analysis were conducted to explore the influence factors of resilience and the relationship between resilience and anxiety.

**Results:**

The level of resilience was 67.93 ± 12.65. Resilience was positively correlated with hope, optimism and perceived social support, and negatively correlated with perceived stress. Hierarchical linear regression analysis showed that hope (β = 0.386, *P* < 0.01), optimism (β = 0.190, *P* < 0.01) and education (β = 0.175, P < 0.01) were positively associated with resilience. The three variables in combination could explain 48.9% of the total variance in resilience. Higher level of resilience was associated less anxiety symptoms (X^2^ = 39.216, *p* = 0.000); and there was linear trend between resilience level and anxiety level among patients with oral cancer (X^2^ = 35.624, *p* = 0.000).

**Conclusion:**

Patients with oral cancer in China had moderate level of resilience. Hope, optimism and education were positively and significantly associated with resilience, indicating that higher level of hope, optimism and education may improve resilience in oral cancer patients, which in turn may help alleviate anxiety symptoms in patients.

## Background

Oral cancer is a broad term for carcinomas in the oral cavity and oropharynx including the floor of mouth, palate, cheek, lip and parotid gland. It was reported that 354,864 cases of oral cavity cancer are diagnosed worldwide every year, and 177,384 of them would die from the disease [1]. In addition to the common problems such as uncontrollable pain, oral cancer patients may also suffer from poor quality of sleep, side effects of treatment, fear of treatment failure or disease recurrence, post-operative facial deformity and dysfunction, and heavy financial burden of the treatment. All these and other traumatic experiences challenge patients’ physical, mental and emotional coping capacity [2], so the patients are generally expected to have a higher level of psychological distress. Indeed, it was reported that the prevalence of anxiety and depressive symptoms in Chinese head and neck cancer patients was much higher than that of the normal population [3]. On the other hand, however, we may find in clinical work that quite a proportion of oral cancer patients can cope with the disease relatively well. Similar phenomenon exists in other cancer patients. For example, a longitudinal study showed that some breast cancer patients had better psychological adaptation in the course of disease, and the same study discovered that patients with higher level resilience were the ones who could better cope with the adversity [4]. We therefore suspect that resilience, one of the positive psychological resources, might play an important role in the disease adjustment process of these patients.

Resilience is a very important concept in psychology and has been the research subject for many decades. However, despite of the rapid advancement in research on resilience, there is no consensus on its definition. As a result, currently three definitions of resilience exist: quality definition, process definition and outcome definition. In the quality definition, resilience is regarded as a stable quality or characteristic of a person that enables one to thrive in the face of adversity, which varies with context or different life circumstances, time, age, gender, and cultural origin [5]. In the process definition, resilience is thought as a development process of interaction and successful adaptation between individuals and the environment. For example, American Psychology Association describes resilience as the process of adapting well or “bouncing back” in the face of adversity, trauma, tragedy, threats or significant sources of stress (e.g. family and relationship problems, serious health problems or workplace and financial stressors) [6]. Finally, in the outcome definition, resilience is considered a positive result of dealing with stressors flexibly by identifying or developing resources and advantages [7]. Despite of diversity in the definition, it is generally accepted that a resiliency process is life-enriching and stressors or adverse changes can provide growth and increased resilient qualities or protective factors [8].

Research on resilience is important to clinical patient care because many studies, both descriptive [9, 10] and interventional [11–13], have shown that resilience associates with patients’ well-being. For instance, resilience has been found to be positively related to the level of mental health and negatively related to distress [14]. It has also been found important to the quality of life [15]. Surprisingly, however, little information about resilience in oral cancer patients is currently available. Oral carcinoma may differ from malignant tumors of other systems in that the disease and its treatments often involve patients’ appearance change and severe impairment in communication. Such profound and distinct adverse effects and the consequences should be dealt with in a patient-centered, wholistic fashion and tackled from different perspectives including psychological methods. We therefore aim to fill the knowledge gap with the current study. Furthermore, studies focusing on the relationship between resilience and other psychological factors have shown that, in addition to the demographic and clinical characteristics, hope [16], social support [6, 17], stress [18] and optimism [19] were all associated with resilience, but their state of relationship in oral cancer patients is unknown. In order to find the predictors of resilience in oral cancer patients, all the above mentioned factors were included in our study. We hypothesize that, in these patients, resilience is positively associated with hope, social support and optimism, respectively, and is negatively associated with perceived stress. We will test the hypothesis in the current study accordingly. In addition, since anxiety is one of the most common psychological problems among cancer patients [20] and may negatively impact the patients’ quality of life, we also hope to explore what role resilience may play in the development of anxiety in oral cancer patients. We hope that the findings of our study and, in particular, the identification of predictors of resilience may help shed new light on clinical management of oral cancer patients for the purpose of improving patients’ mental health care.

## Methods

### Participants

The study was approved by the Committee on Human Experimentation of China Medical University, and the study procedures were in accordance with the ethical standards. Sample patients were recruited between May 2016 and October 2017 from two medical centers (Stomatology Hospital of China Medical University and Department of Stomatology, Shengjing Hospital of China Medical University). After the patients agreed to participate in this cross-sectional study, they were administrated with questionnaires. Inclusion criteria were as follows: (1) patients were at least 18 years old; (2) had been clinically diagnosed with oral cancers for the first time; (3) were aware of their own diagnosis; (4) their condition was well enough to answer the questionnaires and patients had the ability to accurately answer questions. Exclusion criteria were as follows: (1) patients had psychiatric history or cognitive disorders; (2) were not literate enough to complete the survey; or (3) had other oral diseases or other cancers. Eligible patients were invited to join the study by nurses. Investigators who had received relevant training were responsible for helping the patients read and provide explanation for questionnaire items without any inducement. Of the 231 patients who met the inclusion criteria, 230 completed the questionnaires including 134 men and 96 women.

### Measures

Demographic and clinical characteristics were obtained with a general questionnaire. Demographic characteristics consisted of age, gender, BMI, marital status, education level, monthly income, job status, residence area, smoking and alcohol drinking. Clinical variables included type of treatment, family history of cancer and distant cancer metastasis.

### Measurement of resilience

Patients’ resilience was measured with the Resilience Scale-14 (RS-14) [21]. Each item was answered (graded) against a 7-point Likert-type scale ranging from “strongly disagree” to “strongly agree”. The total score of the scale was calculated to obtain a composite resilience value, and higher scores indicated higher levels of resilience. The level of resilience was considered low when the score was ≤63 [22]. In analyzing the correlation between resilience and anxiety, level of resilience was divided into three categories (low, moderate and high) according to its score. The resilience fell to the low category when the score ≤ 63, moderate when the score was 64–73, and high when the score was≥74. The Chinese version had been used in previous studies, and the reliability and validity had been confirmed [22]. The Cronbach’s alpha coefficient for the total scale of resilience was 0.929 in the present study.

### Measurement of Hope

The Herth Hope Index (HHI) [23] was used to measure hope in patients. HHI contained 3 subscales: temporality and future, positive readiness and expectancy, and interconnectedness, with a total of 12 items. Each item had 4 response categories from 1 to 4. Higher total scores reflected higher level of hope. The Chinese version of HHI had been used in cancer patients with good reliability and validity [24]. The Cronbach’s α for hope was 0.841 in the present study.

### Measurement of perceived social support

The Multidimensional Scale of Perceived Social Support (MSPSS) [25] was utilized to measure the level of social support. The 12-item MSPSS measured perceived support from three social relationships: family, friends and significant others (e.g. relatives and colleagues). The score of each item was given on a 7-point Likert-type scale in accordance with the patients’ personal experiences, ranging from 1 (very strongly disagree) to 7 (very strongly agree). The total score ranged from 12 to 84, with a higher score indicating higher social support. MSPSS was shown to have good reliability and validity among various Chinese patients [26, 27]. In the present study, the Cronbach’s αcoefficient for the social support scale was 0.928.

### Measurement of optimism

Optimism was measured with the Revised Life Orientation Test (LOT-R) [28]. The scale contained 10 items, including 3 measuring optimism, 3 measuring pessimism and 4 serving as fillers. Respondents rated each item on a 5-point Likert scale with varying degrees of agreement or disagreement. A higher score indicated higher level of optimism. LOT-R showed good reliability and validity among various Chinese patients [27]. The Cronbach’s α for optimism scale was 0.646 in the present study.

### Measurement of perceived stress

Perceived stress was assessed with Perceived Stress Scale-10 (PSS-10) [29]. Responses to the items were graded on a 5-Likert scale from never to very often. The total score ranged from 0 to 40, and higher scores indicated higher level of perceived stress. The Chinese version demonstrated good reliability and validity [30]. The Cronbach’s α for perceived stress scale in present study was 0.833.

### Measurement of anxiety symptoms

The Zung Self-Rating Anxiety Scale (SAS) was designed to record the presence and quantify the severity of anxiety [31]. The item was on a 4-Likert scale from “never” to “always”(1–4). The total raw score ranged from 20 to 80, and the standardized score was represented as int (1.25 × raw score). Higher score indicated more severe anxiety symptoms. The Chinese version demonstrated good reliability and validity [32]. Studies in the Chinese populations showed that the upper limit for the normative populations was a standardized score lower than 50 [33]. For the purpose of analyzing the relationship between resilience and anxiety, anxiety was categorized into three degrees, mild (score < 50), moderate (score 50–59) and severe (≥60). The Cronbach’s α for SAS scale in this study was 0.908.

### Statistical analyses

Statistical analyses were conducted with the Statistical Package for the Social Science (SPSS, version 17.0). Significance for all statistical tests was set at the level of 0.05 or less (2-tailed). Normality and homogeneity of variances were first tested for each continuous variable. The study used one-way ANOVA/ t-test to describe distributions of resilience in categorical demographic and clinical variables. Correlations between resilience, hope, social support, optimism and perceived stress were conducted using Pearson’s r. Hierarchical linear regression analyses were conducted to test the study hypotheses. Demographic variables that had statistical significance in one-way ANOVA/ t-test were entered into step 1 of the hierarchical regression analysis as control variables. The independent variables (hope, optimism, perceived social support and perceived stress) were entered into step 2. Variables were entered in the regression analysis at *P* < 0.05 and removed from the model at *P* > 0.10. Data provided in the regression models included standardization regression coefficient (β), R^2^, adjust R^2^ (Adj.R2), R^2^-change and F value. Nonparametric tests, Kruskal-Wallis H test and Cochran-Mantel-Haenszel test, were used to test the relationship between level of resilience and level of anxiety.

## Results

### Descriptive statistics and bivariate correlations

In the study, of the 231 distributed questionnaires, 230 were well completed, resulting in an effective response rate at 99.57%.

Demographic and clinical characteristics of the participants and the level of resilience in different categories of variables were described in Table 1. Of the 230 patients, 134 (58.3%) were men and 96 (41.7%) were women. Mean age was 55.47 years (SD = 13.78, range 18–92).

**Table 1:** Demographic and clinical characteristics and the level of resilience among oral cancer patients (*n* = 230)

Variables	N (%)	Resilience		
		Mean (SD)	T /F	P
Age			−0.010	0.992
< 60 years	156 (67.8)	67.93 (13.04)		
≥60 years	74 (32.2)	67.95 (11.87)		
Gender			− 0.743	0.458
Male	134 (58.3)	67.39 (12.58)		
Female	96 (41.7)	68.67 (12.71)		
Marriage			2.069	0.047
Single/divorced /widowed	26 (11.3)	67.41 (12.84)		
Married/cohabitation	204 (88.7)	72.00 (10.37)		
BMI			1.018	0.363
<18.5	8 (3.5)	61.75 (22.62)		
18.5–23.9	118 (51.3)	67.96 (12.10)		
≥24	104 (45.2)	68.36 (12.26)		
Education			3.493	0.032
Middle school or lower	100 (43.5)	67.26 (11.75)		
High or secondary school	60 (26.1)	65.42 (14.18)		
College or university	70 (30.4)	71.01 (11.97)		
Job status			0.524	0.586
Regular employee	133 (57.8)	67.71 (12.73)		
Retirement	34 (14.8)	66.47 (11.58)		
Unemployed /temporary workers	63 (27.4)	69.14 (12.93)		
Income			−1.963	0.051
< 3000	141 (61.3)	66.64 (12.34)		
≥3000	89 (38.7)	69.97 (12.93)		
Residence			1.567	0.119
Urban	145 (63.0)	68.92 (12.57)		
Rural	84 (37.0)	66.22 (12.62)		
Smoking			1.543	0.124
No	118 (51.3)	69.17 (13.16)		
Yes	112 (48.7)	66.61 (12.00)		
Drinking alcohol			0.960	0.338
No	135 (58.7)	68.59 (12.60)		
Yes	95 (41.30)	66.97 (12.67)		
Type of treatment			0.465	0.643
Radical surgery	210 (91.3)	68.33 (12.75)		
Alleviative treatment	20 (8.7)	66.89 (10.32)		
Family history			1.053	0.293
No	215 (93.5)	68.17 (12.69)		
Yes	15 (6.5)	64.60 (12.30)		
Distant metastasis			2.717	0.007
No	216 (94.0)	68.61 (12.16)		
Yes	14 (6.0)	58.67 (15.28)		

Of all the variables, levels of resilience were found significantly different among different categories of the variables including marriage (t = 2.062, *p* = 0.047), education (F = 3.493, *p* = 0.032) and distant metastasis (t = 2.717, *p* = 0.007), whereas the difference in other variables were not statistically significant.

### Correlation among continuous variables

Results of correlation analysis among hope, optimism, perceived social support, perceived stress and resilience were presented in Table 2. Resilience was positively associated with hope (r = 0.656, p<0.01), optimism (r = 0.541, p<0.01) and perceived social support (r = 0.535, p<0.01), and negatively correlated with perceived stress (r = − 0.479, p<0.01).

**Table 2:** Descriptive statistics and correlations among continuous variables (n = 230)

	Means	SD	Resilience	Hope	Social support	Optimism
Resilience	67.93	12.65	1			
Hope	36.43	4.633	0.656**	1		
Perceived social support	60.17	11.34	0.535**	0.678**	1	
Optimism	15.81	2.90	0.541**	0.599**	0.509**	1
Perceived stress	16.67	4.84	−0.479**	−0.580**	−0.365**	−0.536**

**P < 0.01 (two-tailed)

### Hierarchical linear regression analysis

Hierarchical linear regression analysis was conducted to identify the predictors of resilience. Variables that were significantly associated with resilience in the univariate analyses were included in the multiple regression analysis. They were demographic variables (education and marriage), clinical variables (distant metastasis), hope, optimism, perceived social support and perceived stress. Results of the analysis were shown in Table 3. Hope (β = 0.386, *P* < 0.01), optimism (β = 0.190, P < 0.01) and education (β = 0.175, P < 0.01) were found positively associated with resilience, and all the three variables in the model could explain 48.9% of the variance in resilience with psychological variables alone accounting for 42.8%. On the other hand, marriage, distant metastasis, perceived social support and perceived stress showed no significant relations with resilience.

**Table 3:** Hierarchical linear regression analysis on results of resilience (*n* = 230)

Variables	Resilience			
	β	P	β	P
Step 1				
Marriage	−0.086	0.194	−0.008	0.836
Education				
Dummy_1	−0.040	0.569	0.038	0.480
Dummy_2	0.165	0.022	0.175^***^	0.001
Distant metastasis	−0.197	0.003	−0.016	0.756
Step 2				
Hope			0.386^***^	0.000
Optimism			0.190^***^	0.004
Social support			0.119	0.090
Perceived stress			−0.120	0.061
F	4.707^***^		27.793^***^	
R^2^	0.079		0.507	
adjR^2^	0.062		0.489	
R^2^-change	0.048		0.428	

***P < 0.001 (two-tailed)

### The relationship between levels of resilience and anxiety

The relationship between resilience level and anxiety was tested to describe the importance of resilience. Kruskal-Wallis H test was used to determine whether there was statistically significant difference in anxiety among patients with different levels of resilience. In general, 37.0% (85/230) of patients reported anxiety symptoms. As showed in Table 4, levels of anxiety differed among different levels of resilience, and the rate of anxiety for patients with relatively low, moderate and high level of resilience was 62.8, 30.1 and 17.7%, respectively (X^2^ = 39.216, *p* = 0.000). The Cochran-Mantel-Haenszel test was used to clarify the linear variation tendency between resilience and anxiety, and it showed there was a linear trend between resilience level and anxiety level among patients with oral cancer (X^2^ = 35.624, *p* = 0.000).

**Table 4:** Degree of anxiety among patients with different levels of resilience (n = 230)

Level of resilience (n)	None (%)	Mild anxiety (%)	Moderate anxiety (%)	Severe anxiety (%)
≤63 (78)	29 (37.2)	26 (33.3)	18 (23.1)	5 (6.4)
64–73 (73)	51 (69.9)	15 (20.5)	7 (9.6)	0 (0.0)
≥74 (79)	65 (82.3)	10 (12.7)	4 (5.1)	0 (0.0)

Kruskal-Wallis H test: X^2^ = 39.216, p = 0.000;

Cochran-Mantel-Haenszel test: X^2^ = 35.624, *p* = 0.000.

## Discussion

In this study, we first investigated the association of demographic and clinical variables with resilience and we found that education, married/cohabitaton and distant cancer metastasis were the associated factors with resilience. We then did the correlation study and found that Chinese oral cancer patients had moderate level of resilience. Moreover, hope, optimism and perceived social support positively correlated with resilience while perceived stress was negatively correlated. Further study with hierarchical linear regression analysis showed that hope, optimism and education were predicative factors for resilience. Finally, we were able to reveal that resilience was associated with anxiety symptoms in oral cancer patients.

### Resilience level

Our findings regarding the level of resilience (67.93 ± 12.65) are significant due to the lack of information available in the literature about resilience in oral cancer patients. A previous report in China showed that the level of resilience in oral cancer patients was 62. 68 ± 19.47, but the sample size of that report was smaller (110 cases) and the study used a different scale [34]. Our result together with another finding in this study (that higher level of resilience correlated with less anxiety symptoms) may partially explain the relatively high adaptation to the disease by some oral cancer patients despite of the grim nature of the disease. In this respect, it is interesting to note that studies have shown that the implementation of resilience related intervention could increase an individual’s adaptation to frustration and setbacks [14, 35, 36]. Therefore, our findings may provide the basis for future intervention research to improve mental health in these patients.

### Factors associated with resilience

Many potential influencing factors of resilience were considered in this study. However, most of the demographic and clinical variables such as age, gender, BMI, job status, income, residence area, smoking, drinking alcohol, type of treatment and family history of cancer were not related to resilience in the univariate analyses, so these variables were not adjusted in the following hierarchical linear regression analyses. Regression analyses for marriage, education, distant metastasis as well as the psychosocial factors revealed hope, optimism and education as the main predictors of resilience. These three factors in combination explained 48.9% of the variance in resilience of oral cancer patients, with self-reported hope making the largest predictive contribution. To our knowledge, we are the first to report such findings in oral cancer patients. These results may have useful implications for clinical research and management of oral cancer.

The finding that hope had the strongest association with resilience (β = 0.386, *p* < 0.01) was similar to the results in previous studies among other cancer patients. For example, in a longitudinal study, hopefulness was found capable of predicting resilience among hereditary colorectal cancer patients [37]. Hope was also found positively related to the level of resilience among patients with breast cancer, metastatic colorectal cancer and adolescent or young adults with cancer in cross-sectional studies [38–40]. A cancer diagnosis is a stressful event for most individuals. Besides the overload of physical stress caused by cancer and its treatment, many patients experience mental stress such as worries about the prognosis of treatments, disruption of ordinary life functions and the length of survival [41]. Cancer diagnosis and its subsequent treatment decreased patients’ hope level and increase psychological distress [42]. In this sense, our finding that hope was the strongest predictor for resilience in oral cancer patients is important, because hope is a positive psychological resource for people experiencing difficult situations. It was reported that hope could give cancer patients reasons for survival [43]. Study also demonstrated that hope was negatively associated with negative affectivity and positively related to life satisfaction in newly diagnosed cancer patients [44]. Therefore, enhancing the level of hope may become one of the important strategies to increase the level of resilience among oral cancer patients.

In addition to hope, we found that optimism was another positive psychological resource for resilience (β = 0.190, *p* < 0.01). This result was also consistent with the previous findings by others. For instance, one study demonstrated that individuals with lower optimism were more adept to engaging cognitive and behavioral resources to promote resilience; optimism and resilience synergistically promoted adjustment to chronic pain, making patients feel less severe pain [19]. Optimism is a mental attitude of positive expectation or belief on things that are about to happen. It is a positive and open-minded attitude towards life, which can improve patients’ life satisfaction, reduce negative emotions and behaviors, and improve their quality of life. All the features of optimism are favorable to person’s resilience. Importantly, a study found that, although optimism was a stable personality trait, cognitive behavioral therapy could convert pessimism to optimism through certain activities including encouraging individuals to take part in positive social activities, to feel positive living atmosphere, and reducing the intensity of stressors [45]. Therefore, we suggest that optimism should be included in clinical psychological counseling, and clinicians should help patients develop optimism to adjust their psychological pressure and relieve the negative emotions.

In this study, we also found that education level made a limited predictive contribution as indicated by a modest positive relationship between education and resilience (β = 0.175, *p* < 0.01). The influence of education on patients’ psychology had been confirmed in many studies. Some researchers even found educational level a direct predictor of resilience [45, 46]. This should be easy to understand as higher level of education endows a person stronger ability to acquire knowledge on health and the awareness of methods to combat illness. In addition, people with higher level of education may obtain a better income that would make him or her feel more hopeful of completing the treatment.

On the other hand, the finding in this study that perceived social support and perceived stress were not statistically significant factors for resilience were not consistent with previous researches [45, 47]. Although this phenomenon was somewhat surprising, it should not be so difficult to understand. Resilience may not be significantly affected by things of short duration or sudden, because it is the inner strength of a person himself or herself. This strength or positivity is formed from inherent quality as well as long term accommodation to the environment/atmosphere. In our sample population, all the three factors i.e. education, optimism and resilience are all internal factors or factors that had been developed before the disease was diagnosed. This phenomenon together with the new understanding of resilience reminds us that we should carry out further studies on the clinical use of the psychological resource scales or other scales to screen patients for lower education and psychological capitals so that interventions may be taken to better care patients with low level of resilience.

### The relationship between level of resilience and level of anxiety

In the present study, we found that the levels of anxiety varied with different levels of resilience (X^2^ = 39.216, *p* = 0.000). Patients with higher resilience reported lower level of anxiety. The study also showed that there was a linear trend between resilience level and anxiety level among patients with oral cancer (X^2^ = 35.624, p = 0.000). This finding indicated that the level of anxiety among oral cancer patients decreased as the resilience was improved. As one of the main forms of psychological disorders suffered by cancer patients, anxiety had been studied in many researches [48–50], and it was all agreed that high prevalence of anxiety among the cancer patients necessitated an adequate management in addition to the traditional cancer therapy. In this sense, our study is important because it for the first time demonstrated the positive role of resilience in anxiety symptoms of oral cancer patients, which indicates that improving resilience may have the potential to alleviate anxiety in these patients as well. Therefore, methods should be sought to increase the level of resilience in order to improve the mental status of oral cancer patients.

An important value of this study is that we have identified the potential influencing factors associated with resilience in oral cancer patients for the first time. In this respect, our study has added to the evidence that hope, optimism and education are important factors for individuals with oral cancer, and may partially explain why individuals have different levels of resilience. Skinner’s reinforcement theory holds that behavior is a function of outcome, that is, the production of behavior is affected by certain results. If the result of a behavior is pleasant, it will increase the behavior, and this is called positive reinforcement. Psychological interventions that aim to increase patients’ social, spiritual and psychological well-being are important parts of the multidisciplinary approach to the treatment of oral cancers. Psychological interventions have been developed with a variety of modality that includes health education, psychotherapy, cognitive behavioral therapy, and supportive and group interventions. With the development of positive psychology, interventions based on positive psychology also have been reported in many studies. Our study further demonstrated the positive strengths for oral cancer patients to combat severe diseases and indicated that intervention strategies to improve level of resilience through rebuilding and enhancing the level of resilience should be considered for health care organizations. Up to now, psychological nursing or counseling strategies in clinical settings have not been enough. Therefore, it is necessary to explore the influencing factors of resilience in patients with oral cancer in order to develop strategies.

Nevertheless, there are some limitations in this study. First, because of the cross-sectional design, the causal relationship couldn’t be confirmed. Future researches should be carried out to assess whether interventions could improve the level of resilience among patients with oral cancer. Second, we only focused on the associations of resilience with hope, perceived stress, social support and optimism, while other factors which might be important to consider for resilience were not included. Moreover, a larger and multicenter sample is still needed to improve the representativeness of the findings. Despite of the limitations, our study does provide important new information on resilience in oral cancer patients and it has useful theoretical and clinical implications. It also suggests that an interventional strategy with positive factors may be beneficial to the patient care.

## Conclusion

Our study showed Chinese oral cancer patients had moderate level of resilience; hope, optimism and education were predictive factors for resilience. In addition, we found that higher level of resilience in oral cancer patients was associated with lower level of anxiety. These results suggest that interventions that enhance hope and optimism in oral cancer patients might help promote their resilience, which in turn may help improve the patients’ mental distress such as anxiety.

## Abbreviations

ANOVA: Analysis of variance
BMI: Body mass index
HHI: Herth Hope index
LOT-R: Revised life orientation test
MSPSS: Multi-dimensional scale of perceived social support
PSS-10: Perceived stress scale-10
RS-14: Resilience scale-14
SAS: Zung self-rating anxiety scale
SD: Standard deviation
